# Exploring Non-puerperal Uterine Inversion: A Case Series

**DOI:** 10.7759/cureus.53071

**Published:** 2024-01-27

**Authors:** Ramanujam Shasindran, Nadaraja Dharshini, Naidu Aruku, Arajunan Sasikala

**Affiliations:** 1 Obstetrics and Gynecology, Hospital Raja Permaisuri Bainun, Ipoh, MYS

**Keywords:** vaginal hysterectomy, non-puerperal, hultain's procedure, ocejo’s technique, uterine inversion

## Abstract

Uterine inversion is a condition characterized by the turning of the uterus inside out. We present a case series of non-puerperal uterine inversion that we managed based on the clinical presentation. A total of three cases highlight the challenges associated with diagnosis and surgical management, respectively. As a side note, we also have reviewed the available literature regarding the type of management available.

## Introduction

Uterine inversion is a rare medical condition that involves the inversion or turning inside out of the uterus. In 1951, Jones classified uterine inversion into two distinct types: puerperal/obstetrics and non-puerperal/gynecological [1,2]. The former is associated with childbirth occurring in the postpartum period, while the latter is unrelated to pregnancy, manifesting in non-obstetrical settings. Further categorization of uterine inversion involves a multi-stage description. In Stage 1, the inversion is intrauterine or incomplete, with the fundus remaining within the uterine cavity. In Stage 2, there is a complete inversion of the uterine fundus through the cervix within the vagina. Stage 3 marks total inversion, wherein the fundus protrudes through the vulva. In Stage 4, the vagina becomes involved, resulting in complete inversion through the vulva, accompanied by an inverted uterus [3].

Non-puerperal uterine inversion is a rare entity. The existing literature reports approximately 170 cases with prolapsed uterine fibroid identified as the most common cause [4]. This rarity and the condition's atypical presentation contribute to clinical challenges. The symptoms include pelvic pain, vaginal bleeding, and the presence of a palpable mass, are nonspecific and can mimic other gynecological conditions. This condition often results in delayed diagnosis, contributing to the complexity of treatment decisions. The management requires a highly individualized approach due to the varied clinical presentations and causes. Chronic uterine inversion typically necessitates elective surgery, and the choice of surgical method depends on various factors [1]. Our case series aims to contribute valuable insights into the diagnostic and surgical challenges encountered and the approach to managing the challenges of uterine inversion.

## Case presentation

Case 1

A 54-year-old lady with underlying hypertension, diabetes mellitus, and bronchial asthma was referred to our center for prolapse submucosal fibroid. She presented with heavy menstrual bleeding for two years and required a blood transfusion for anemia. A local examination showed a normal vulva; however, the patient refused a vaginal examination. A transabdominal ultrasound revealed a heterogeneous mass originating from the uterus. A computerized tomography scan revealed a lobulated heterogenous enhancing mass with central hypodensity arising from the uterine body measuring 5.3x5.3x5.2cm with another lobulated heterogenous mass arising from the lower vagina measuring 4.1x3.6x3.6cm. The patient was suspected to have submucosal fibroid and was offered examination under anesthesia. The patient refused further treatment and insisted on conservative management.

**Figure 1:**
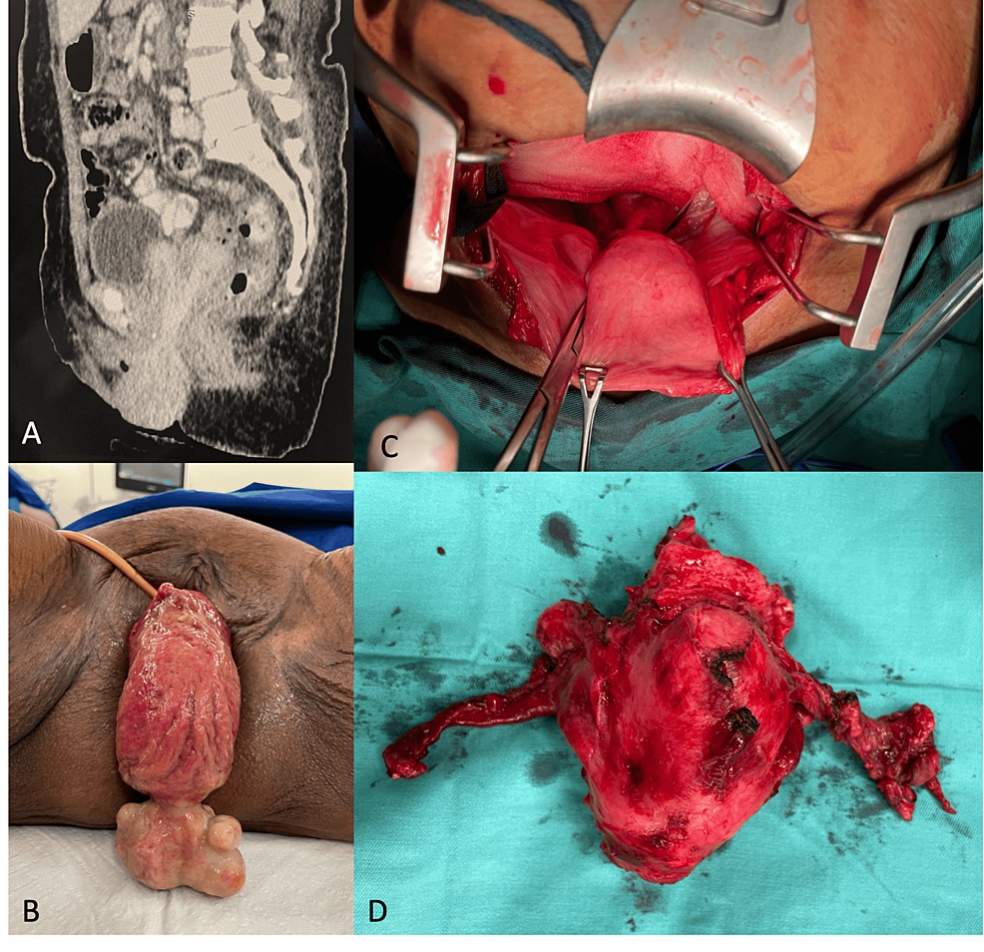
(A) Computerized tomography scan showing partial uterine inversion not protruding beyond introitus, (B) inverted uterus with infected prolapsed submucosal fibroid, (C) exploratory laparotomy showing bladder with cup-like depression over mid-pelvis confirming uterine inversion, (D) postoperative specimen.

The patient returned to our center after four months with complaints of dizziness, mass per vagina, and heavy menstrual bleeding. On history, the mass per vagina was evident on squatting and reducible. In comparison with the abdominal scan and previous computerized tomography scan, a diagnosis of uterine inversion was made (Figure 1A). On examination, the patient had pallor. Her vital signs were within normal limits. Abdominal examination was unremarkable. Vaginal examination revealed an inverted uterus 8x6cm with fundal attachment of submucosal fibroid measuring 4x4cm that appeared infected (Figure 1B). The cervical rim was edematous and ill-define. The vaginal length was about 4cm. The transabdominal scan showed pelvic cavity indentation at the lower part of the bladder without a visible uterus and normal kidneys. We organized a multidisciplinary team meeting with the radiologist.

Blood investigations were within normal range. We conveyed the diagnosis and course of management to the patient. After shared decision-making, we planned a Haultain's procedure followed by extra fascial hysterectomy and bilateral salphingo-oophorectomy.

The patient was scheduled for an emergency procedure in the theatre. The patient was given general anesthesia and positioned in lithotomy. Exploratory laparotomy with pfannensteil skin incision was performed a cup-like depression was identified within the pelvic cavity confirming the diagnosis (Figure 1C). We approached the patient vaginally, and a myomectomy was performed. With the aid of an assistant at the vaginal end, the inverted uterus was manually reduced till the level of constriction ring. The uterovaginal fold was identified, and the bladder was dissected away. Haultain's procedure was abandoned due to difficulty identifying the posterior constriction ring. Ocejo's technique was used by incising the anterior aspect of the constriction ring, and the uterus was reverted to its normal position. Myometrium and endometrium were opposed before extra fascial hysterectomy with bilateral salphingo-opherectomy. The estimated blood loss was about 600 mL. The patient was transfused with one pint of blood. The patient recovered well and was discharged with a urinary catheter. A trial without a catheter was scheduled after one week, and the patient could micturate. The six-week review post-operation was unremarkable. Histopathological examination findings showed a benign uterus ovaries and fallopian tube with an infected submucosal fibroid.

Case 2

A 76-year-old lady, para three, presented to the district hospital with acute urinary retention and mass per vagina. She experienced incomplete voiding with poor stream for a few months before her visit. Her vitals were normal, and her abdominal examination was unremarkable. Pelvic examination revealed an irreducible vaginal mass that appear gritty with the absence of the cervix. A Foley catheter (size 10) was cautiously inserted to alleviate the urinary retention. A pelvic ultrasound reported multiple bladder calculi with an absent uterus and a normal kidney. Microscopic urine examination was positive for erythrocytes and leucocytes. Blood parameters were within normal range.

**Figure 2:**
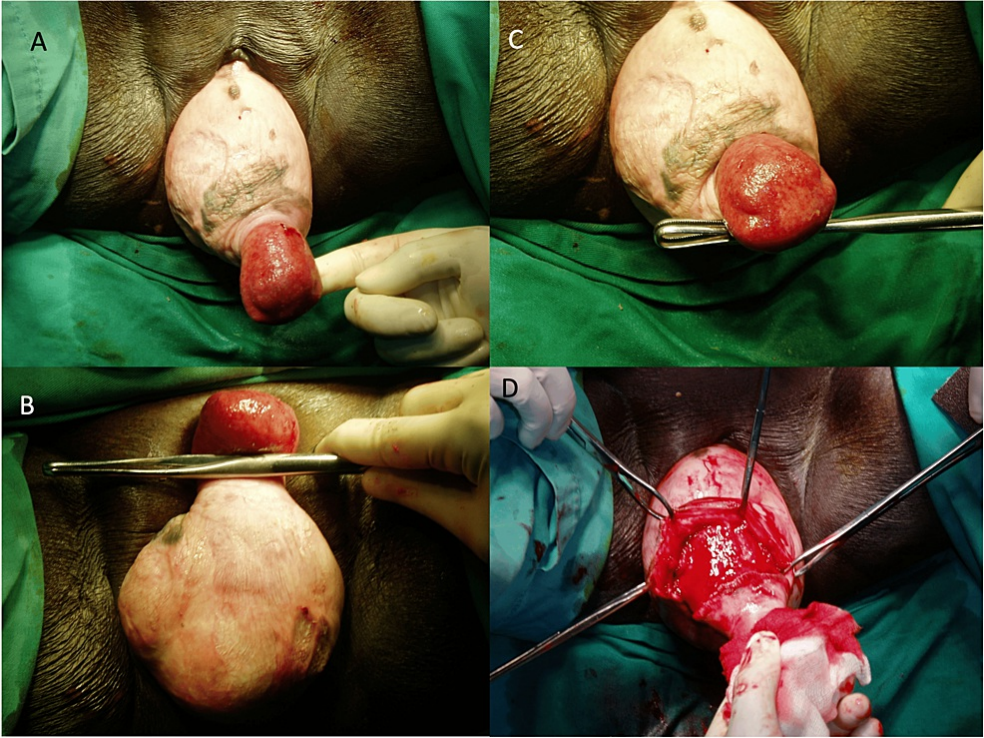
(A) Uterine inversion with grade 3 anterior vaginal wall prolapse, (B) inverted uterus with bilateral ostium and fundus, (C) inverted uterus with large enterocele, (D) vaginal hysterectomy.

Examination under anesthesia revealed a stage 3 cystocele with uterine inversion mimicking a procidentia (Figure 2A). Bilateral ostia was identified at the distal end of the prolapsed mass (Figure 2B). The cervix was within the vagina. Cystoscopy examination was not feasible as multiple bladder stones obscured the bladder. A diagnosis of chronic uterine inversion with stage 3 cystocele and bladder calculi was made. The patient was given the diagnosis and plan of action, and a shared decision was made. A multidisciplinary team brief with the urology team helped with the course of management. The planned procedure was the repositioning of the uterus followed by a vaginal hysterectomy, cystolithotripsy, cystolithotomy, bladder repair, anterior repair, and colpocleisis.

The patient was scheduled for an elective procedure after one week. Four longitudinal incisions were made over the constricted band tissues around the inverted uterus and cervix (a combination of the Spinelli and Kustner technique). This procedure aided with the repositioning. A vaginal hysterectomy was carried out (Figure 2D). Cystolithotripsy was abandoned because of the bladder calculi, and the approach moving forward was a transvaginal cystolithotomy. A vertical incision was made on the cystocele, avoiding the bladder neck. We removed 30 sandy materials of variable sizes, from a few millimeters to the largest stone measuring 2.5x3.0cm. The bladder was irrigated with normal saline and repaired in two layers. A check cystoscopy was carried out to identify ureteric orifices for injury and flow of urine. The incision and stitch were at the supratrigonal region. A methylene blue test was performed, and leakage was ruled out. Anterior colporrhaphy was performed and opposed with pubocervical fascia. A total colpocleisis was performed.

The patient recovered well and was discharged with a bladder catheter. A trial without a catheter was scheduled after two weeks. The cystogram was normal. She was reviewed at six weeks, showing good recovery. Histopathological examination revealed a benign uterus. The patient was followed up at three and six months before being discharged from our center.

Case 3

A 61-year-old woman, para two, with underlying hypertension, was referred to our center with abdominal pain and mass per vagina. The patient complained of severe pain, unrelieved by potent analgesics. The abdomen was soft on examination, with vague tenderness over the bilateral iliac crest.

The patient was reluctant to undergo a vaginal examination. After discussion and reassurance, the vaginal examination was performed. Vaginal examination revealed uterine inversion, 5cm from introitus, with prolapse submucosal fibroid measuring about 5x5 cm. The transabdominal scan showed pelvic cavity indentation at the lower part of the bladder without a visible uterus and normal kidneys. Blood parameters were within normal limits.

The diagnosis and shared decision-making on the management were conveyed to the patient. An abdominal approach by Haultain procedure followed by an extra fascial hysterectomy and bilateral salphingo-oopherectomy was planned by us.

**Figure 3:**
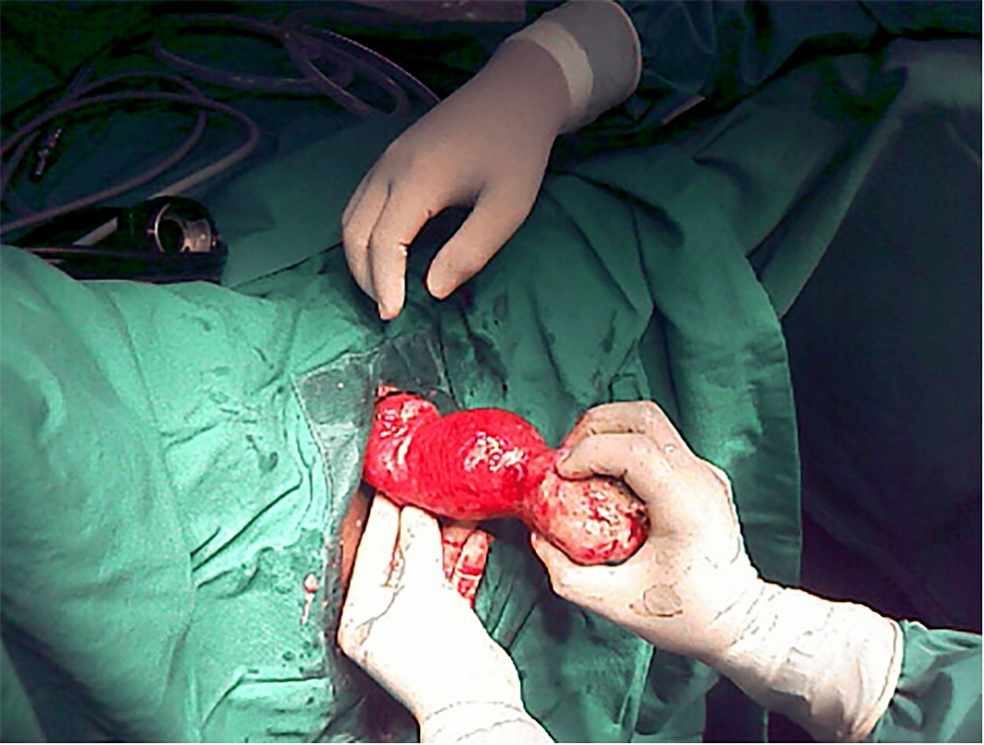
Prolapsed inverted uterus with fundal fibroid.

The patient was scheduled for an emergency procedure in the theatre. The patient was under general anesthesia and placed in the lithotomy position. Exploratory laparotomy with midline skin incision was performed cup-like depression was identified within the pelvic cavity confirming the diagnosis. With the help of the assistant at the vaginal end, a myomectomy was done, and the uterus was manually repositioned (Figure 3). Abdominally, the uterovaginal fold was identified, and the bladder was dissected away. The constriction ring was identified in the anterior and posterior aspects. The Haultain procedure was performed by incising the posterior aspect of the constriction ring, and the uterus was returned to its normal position. An extra fascial hysterectomy with bilateral salphingo-opherectomy was performed. The surgery was successful. The estimated blood loss was about 300mls. Postoperatively, the patient was well and discharged with a urinary catheter. A trial without a catheter was scheduled after one week, and the patient could micturate. Six weeks' review was unremarkable. Histopathological examination findings showed benign uterus ovaries, fallopian tubes, and submucosal leiomyoma.

## Discussion

Non-puerperal uterine inversion poses significant challenges in terms of diagnosis and management. In the literature, cases of non-puerperal uterine inversion have been reported in association with various underlying pathologies, such as uterine fibroids, polyps, cervical tumors, and pelvic masses [3].

The diagnostic process can be complicated as uterine inversion can be mistaken for other gynecological conditions such as pelvic tumor and pelvic organ prolapse. A clinical suspicion and a meticulous physical examination are critical for overcoming this diagnostic hurdle.

Imaging modalities such as transabdominal or transvaginal ultrasound, magnetic resonance imaging (MRI), and computed tomography (CT) scans are valuable tools for visualizing the inverted uterus and identifying the underlying cause. However, these modalities have potential limitations, such as their dependence on operator expertise and the inability to provide real-time information. On a sagittal image, a U-shaped uterine cavity with a thickened, inverted uterine fundus is a sign to confirm uterine inversion [5]. The imaging findings must be interpreted with clinical judgment to avoid misdiagnosis.

The management of non-puerperal uterine inversion is contingent upon several factors, including the underlying cause, the patient's overall health, and the severity of symptoms. Options range from conservative measures to surgical intervention. Conservative measures are considered in hemodynamically stable patients and when the mass can be manually reduced. This approach involves attempting to reposition the inverted uterus without resorting to surgery. While conservative measures can be effective in selected cases, their success hinges on factors such as the patient's anatomy, the underlying cause of the inversion, and the promptness of intervention. The author believes after the reduction of the inversion, a space-occupying pessary may aid in the prevention of recurrence.

Surgical intervention becomes imperative in cases where conservative measures are unsuccessful or inappropriate. Hysterectomy is the treatment of choice for non-puerperal uterine inversion, except in women who desire fertility-sparing [6,7]. The different surgical approaches are highlighted in Table 1 [8,9].

**Table 1:** Choices of surgical approach

Procedure	Techniques
Vaginal approach	
Spinelli technique	The incision is made on the anterior aspect of the cervix, and then the uterus is repositioned
Kustner technique	Enter the pouch of Douglas vaginally and split the posterior aspect of the uterus and the cervix for reinverting the uterus
Abdominal approach	
Haultain's technique	Approximately 1.5 inches of incision is made in the posterior surface to transect the constriction ring.
Ocejo's technique	Incision made over the anterior surface of the incision are usually avoided to reduce the risk of accidental cystotomy.
Huntington technique	A clamp is placed on each round ligament or myometrium. Gently pull on the clamps to exert upward traction on the inverted fundus. Repeat it until the inversion is corrected.
Tjalma's technique	Perform abdominal retroperitoneal dissection of ureters and uterine arteries before progressing to hysterectomy after opening the vaginal wall anteriorly.

In this case series, we have used multiple techniques as described above. Hultain's procedure appears to be the most successful method to achieve repositioning in a literature review [2]. The choice between vaginal or abdominal approaches is not straightforward and depends on various factors, including the surgeon's expertise, the patient's anatomy, and the specific characteristics of the inverted uterus [10]. Factors such as the presence of concomitant pelvic pathology, the desire for future fertility, and the overall health of the patient should guide the decision-making process. The surgeon must weigh the advantages and disadvantages of each method [4]. Vaginal procedures may be technically challenging in cases where the uterus is non-prolapsing.

Additionally, the potential for intra-operative complications, such as bleeding or injury to surrounding structures such as the ureter, bladder, and bowel, underscores the importance of surgical experience and expertise. Postoperative care is equally crucial for ensuring optimal outcomes. Monitoring for complications, such as infection or recurrence of inversion, requires vigilant postoperative management.

## Conclusions

Non-puerperal uterine inversion is a rare condition that poses challenges in both diagnosis and management. Its nonspecific symptoms make it difficult to identify, and as we continue to gain a better understanding of its underlying causes, there is a need for further research to refine diagnostic criteria and explore novel imaging modalities. Treatment options depend on various factors, including the underlying cause and the patient's overall health, and may include conservative measures or surgical intervention. Surgical management can be effective but requires careful consideration and ongoing monitoring. Advancements in imaging technology, such as three-dimensional ultrasound and laparoscopy, show promise in improving the accuracy and efficiency of diagnosis, offering real-time visualization and helping surgeons navigate the complexities of non-puerperal uterine inversion. Further research and collaboration are essential for advancing our understanding of this condition and optimizing diagnostic and treatment approaches.
